# The incidence and mortality of yellow fever in Africa: a systematic review and meta-analysis

**DOI:** 10.1186/s12879-021-06728-x

**Published:** 2021-10-23

**Authors:** Akuoma U. Nwaiwu, Alfred Musekiwa, Jacques L. Tamuzi, Evanson Z. Sambala, Peter S. Nyasulu

**Affiliations:** 1 Division of Epidemiology & Biostatistics, Faculty of Medicine & Health Sciences, Stellenbosch University, Cape Town, South Africa; 2 School of Health Systems & Public Health, Faculty of Health Sciences, University of Pretoria, Pretoria, South Africa; 3 Cochrane South Africa, South African Medical Research Council, Cape Town, South Africa; 4 School of Public Health and Family Medicine, Kamuzu University of Health Sciences, Blantyre, Malawi; 5 Division of Epidemiology & Biostatistics, School of Public Health, Faculty of Health Sciences, University of the Witwatersrand, Johannesburg, South Africa

**Keywords:** Yellow fever, Incidence, Outbreak, Systematic review, Meta-analysis, Africa

## Abstract

**Background:**

Understanding the occurrence of yellow fever epidemics is critical for targeted interventions and control efforts to reduce the burden of disease. We assessed data on the yellow fever incidence and mortality rates in Africa.

**Methods:**

We searched the Cochrane Library, SCOPUS, MEDLINE, CINAHL, PubMed, Embase, Africa-wide and Web of science databases from 1 January 1975 to 30th October 2020. Two authors extracted data from included studies independently and conducted a meta-analysis.

**Results:**

Of 840 studies identified, 12 studies were deemed eligible for inclusion. The incidence of yellow fever per 100,000 population ranged from < 1 case in Nigeria, < 3 cases in Uganda, 13 cases in Democratic Republic of the Congo, 27 cases in Kenya, 40 cases in Ethiopia, 46 cases in Gambia, 1267 cases in Senegal, and 10,350 cases in Ghana. Case fatality rate associated with yellow fever outbreaks ranged from 10% in Ghana to 86% in Nigeria. The mortality rate ranged from 0.1/100,000 in Nigeria to 2200/100,000 in Ghana.

**Conclusion:**

The yellow fever incidence rate is quite constant; in contrast, the fatality rates vary widely across African countries over the study period. Standardized demographic health surveys and surveillance as well as accurate diagnostic measures are essential for early recognition, treatment and control.

**Supplementary Information:**

The online version contains supplementary material available at 10.1186/s12879-021-06728-x.

## Background

Yellow fever is an acute systemic illness caused by flavivirus transmitted by infected mosquitoes belonging to the *Aedes* and *Haemogogus species* [1]. Most of the yellow fever cases identified in Africa are seen in the unvaccinated population who live in the yellow fever belt. In severe cases, this viral infection causes high fever, bleeding into the skin and death of cells in the liver and kidney [2]. Currently, there is no treatment, cure or drugs for yellow fever but the disease can be prevented through vaccination. A yellow fever vaccine is available and recommended to laboratory workers, children over 9 months and people who are traveling to or living in yellow fever high-risk areas of Africa [3]. A diagnosis of yellow fever is difficult to make as the definition for suspected cases is based on similar signs and symptoms to other diseases like malaria, typhoid, dengue fever and other haemorrhagic fevers. Laboratory diagnosis exists, but the availability and lack of diagnostic capacity are major challenges in African countries. Consequently, it is a major public health problem often underreported in Africa.

**Fig. 1:**
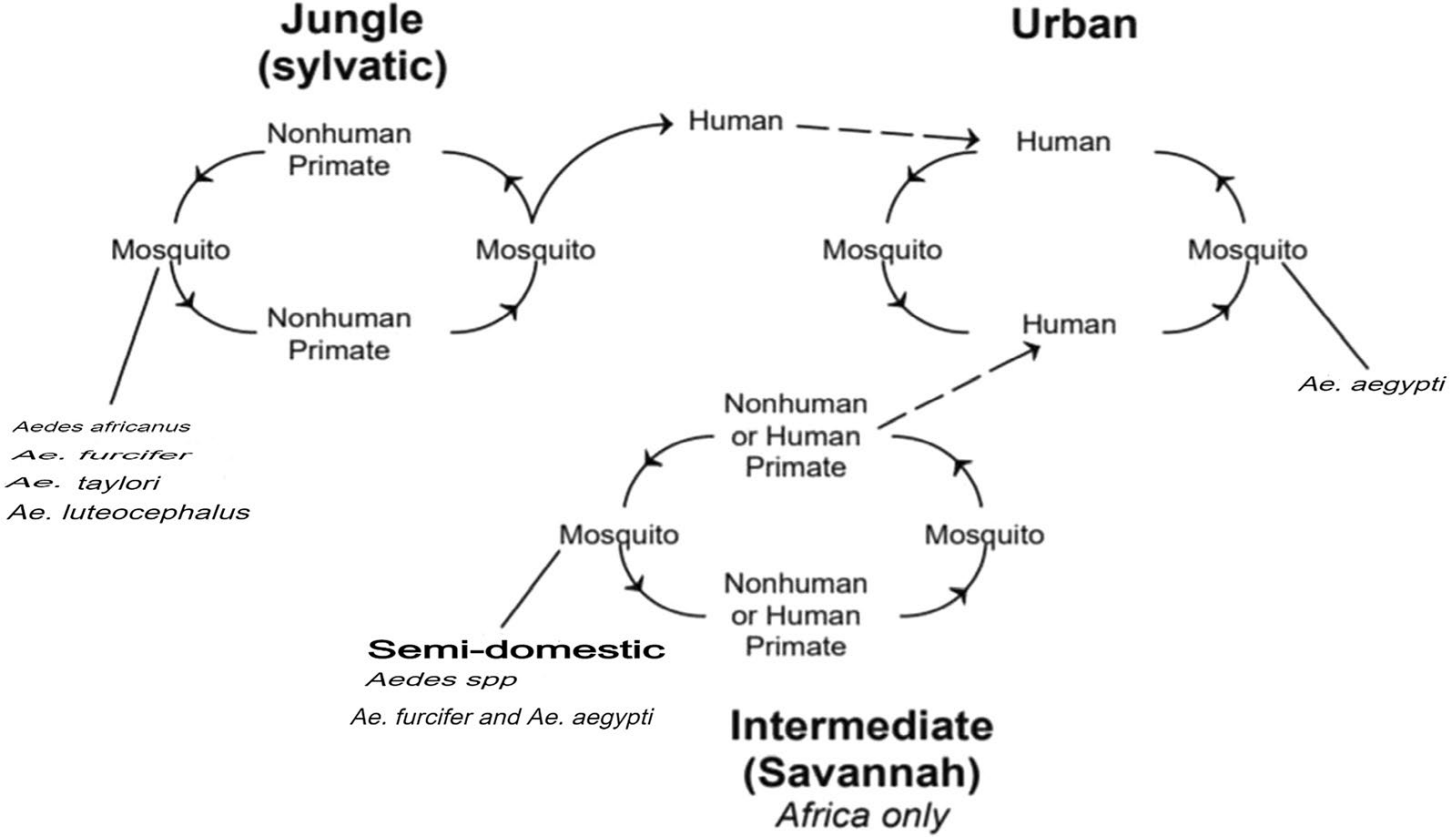
Transmission cycle of yellow fever virus

Yellow fever has three modes of transmission cycles (Fig. 1). The first is sylvatic or jungle yellow fever. It happens when monkeys living in the tropical rainforest are infected through mosquitoes bites; and when humans visit or work in the jungle, the virus is transmitted from monkeys to humans by mosquitoes [4]. Other mosquitos that do not carry the virus become infected when they feed on the blood of the bitten monkeys, and the cycle continues [4]. In Africa, yellow fever is transmitted by five sylvatic vectors among which four are sylvatic (*Ae. africanus*, *Ae. furcifer*, *Ae. taylori* and *Ae. luteocephalus*) and one is intermediate savannah (*Ae. aegypti*) [5].

The second is the intermediate yellow fever (savannah) cycle, which includes the transmission of the virus from mosquitoes to humans living or working in the jungle border areas [4]. In this cycle, the virus can be transmitted from monkey to human or from human to human through mosquitoes [4]. While the third is urban yellow fever, which includes the transmission of the virus between humans and urban mosquitoes, mainly *Ae. aegypti* [6]. The virus spreads in the urban environment through humans who were infected through mosquito bites in the forest or savannah. Outbreaks are typically triggered by sylvatic and intermediate forms where the virus is transmitted when humans come into close contact with monkeys [6].

Yellow fever has a high case fatality rate, and there is no known cure for this disease [6]. Good in-hospital supportive treatment improves survival rate. In Africa and South America, 200,000 cases of yellow fever are recorded every year of which 90% occur in Africa, causing approximately 30,000 deaths [7]. It is estimated that as many as 610 million people in 32 African countries, including more than 219 million dwelling in urban settings remain at high risk of contracting yellow fever [8].

The World Health Organization (WHO) has reported few cases of yellow fever from 1980, until 2016, when an upsurge in cases, to as high as 1040 at highly irregular intervals were recorded compared to cases between 1986 and 1995 [9, 10]. However, it is uncertain whether the recent increase in number of cases is due to increased surveillance or increased disease activity in these countries. Understanding yellow fever epidemiology as determined by its evolution is important to develop preventative measures such as immunization policies to mitigate yellow fever infection. Yellow fever cases are frequently reported in West Africa than anywhere else in the world, followed by East Africa [11]. Major epidemics have occurred in West Africa posing global threats. An historical outbreak first of its’ kind was recorded in Kenya, East Africa, in 1992–1993 [11]. It was reported that two cases of yellow fever were imported to Europe from West Africa in recent years which had fatal outcomes, suggesting intercontinental transmission [11].

Due to lack of reporting of yellow fever cases in Africa, the overall burden of the disease may potentially be underestimated. The WHO estimated the burden of and associated mortality of yellow fever in Africa to be between 84,000–170,000 severe cases and 29,000–60,000 deaths occurred in 2013 [12, 13]. While some estimates of the burden of yellow fever exists in Africa, we could not find a published systematic review and meta-analysis on yellow fever for the entire Africa continent. Thus this review was aimed to quantify and summarize the overall burden of and fatality associated with yellow fever in Africa.

## Methods

### Eligibility criteria

In this review, we considered outbreak reports, cross sectional studies and other observational studies (case series, case report, epidemiological surveys, surveillance studies etc.) including published and unpublished studies carried out in Africa that reported the incidence, mortality and case fatality rate of yellow fever across all age groups. We included only patients who were considered to have suspected or confirmed cases of yellow fever. Suspected cases were defined as cases that were characterized by acute onset of fever followed by jaundice within 2 weeks of the onset of the first symptoms and confirmed cases as suspected cases that were laboratory-confirmed or epidemiologically linked to a laboratory-confirmed cases or outbreaks. Probable cases were defined as a suspected case and one of the following: (i) epidemiologically link to a confirmed case or an outbreak; (ii) positive post-mortem liver histopathology.

We considered probable cases as being confirmed cases when they included any of the following: a probable case and one of the following: (i) detection of yellow fever-specific IgM; (ii) detection of four-fold increase in yellow fever IgM and/or IgG antibody titres between acute and convalescent serum samples, (iii) detection of yellow fever virus-specific neutralizing antibodies.

All the different modes of transmission were considered and both hospital and field patients (population based) were included in this review.

### Search method for identification of studies for inclusion

We exclusively searched the following databases: Cochrane library, MEDLINE, PUBMED, Embasse, SCOPUS, CINAHL (EBSCOhost), Africa-wide (EBSCOhost) and Web of science (SCI-EXPANDED) for studies published from 1 January 1975 to 30 October 2020 including unpublished studies. We selected the starting date of 1975 because of the generally increasing availability and quality of published studies from that date. The combination of keywords was used to search for relevant studies from the electronic databases. We included the following keywords: “outbreak”, “Burden of disease”, Incidence, Prevalence, Survey, Surveillance, Epidemic, Epidemiology, “yellow fever”, “yellow jack”, “yellow fever vaccines”, vaccination”, seroprevalence, “haemorrhagic fever”, “Jungle fever,” “cross sectional studies”. Boolean terms, AND/OR were used during the keywords search to identify relevant studies. Regional grouping such as sub-Saharan Africa and West Africa were also used to look for studies indexed under regional names. Medical Subject Heading (MesH) terms were used in PubMed and Medline search (Additional file 1: Table S1). In consultation with a medical librarian, peer-reviewed journal papers were searched systematically in SCOPUS, Africa-wide (EBSCOhost) and Web of science (SCI-EXPANDED) using variations of MeSH terms (Additional file 1: Table S1). Lastly, we also controlled vocabulary (subject headings) to search CINAHL, and Embase.

We identified three conferences related to yellow fever through a Google search: International Society for Infectious Diseases (ISID), International Congress on Infectious Diseases (ICID) and International Conference on Infectious Disease Dynamics (ICIDD). We searched for the following terms on the website of each of the three conferences identified: yellow fever, incidence, mortality, and Africa. We also consulted an expert librarian at Stellenbosch University, South Africa, to improve and sharpen the search strategy (Additional file 1: Table S1). We identified other eligible studies by searching the reference list of included studies.

### Data extraction

Two review authors independently and in duplicate screened titles and abstracts and selected studies for inclusion in this review using the set eligibility criteria. The identified studies were retrieved for full texts and included in the review after re-screening. Screening and data extraction was done using Covidence manager. Any discrepancies were resolved by consensus or by a third reviewer. We independently and in duplicate extracted the data on the following: burden of disease, characteristics of the participants, study setting, study design, date of study, study location. Risk of bias for each of the included studies was assessed using the validated quality appraisal tool developed by Hoy and colleagues (Additional file 1: Table S3) [14]. We assessed each domain as either low or high risk of bias and regarded studies which were unclear as high risk of bias. We scored the overall risk of bias according to the number of high risk of bias parameters per study: low (1–3), moderate (4–6) and high (7–9).

### Data synthesis and management

All included studies focused on incidence, case fatality rate (CFR), and mortality. We calculated incidence by dividing the number of confirmed and suspected cases by the total population in that region and expressed it per 100,000 populations. We calculated 95% CI for the incidence using the standard formula for calculating the standard error of a proportion, per 100,000, that is,

$$\sqrt{\begin{array}{c}\varvec{p}\left(100,000-{\varvec{p}}\right)\\ \varvec{n}\end{array}}$$

where p = incidence (per 100,000), and n = sample size. We assumed normality of the incidence statistic and used the critical value of 1.96 while calculating the 95% confidence intervals. We calculated CFR by dividing the number of deaths from yellow fever over a defined period of time by the number of individuals diagnosed with yellow fever then multiplied by 100 to yield a proportion. Mortality rate was calculated by dividing the number of deaths by the total population and then multiplied by 100,000.

We performed random-effects meta-analysis due to the variability in incidence estimates from different countries. We assessed heterogeneity using both the Chi-square test (p < 0.10 considered significant) and the I-square test statistic (> 50% considered significant) [15]. We investigated sources of heterogeneity through subgroup analysis with respect to the country of study [14]. Heterogeneity was also explored by examining the potential differences in the characteristics of the population such as the settings and other characteristics in the ‘Characteristics of included studies’ table. We performed meta-analyses using STATA version 15 and displayed results using forest plots. However, due to significant heterogeneity in meta-analyses for incidence rate and CFR, we performed systematic reporting of the results per study per country. The meta-analysis for mortality rate was not possible due to insufficient data; the results for the mortality rate were reported narratively.

## Results

### Identification of studies for review

**Fig. 2:**
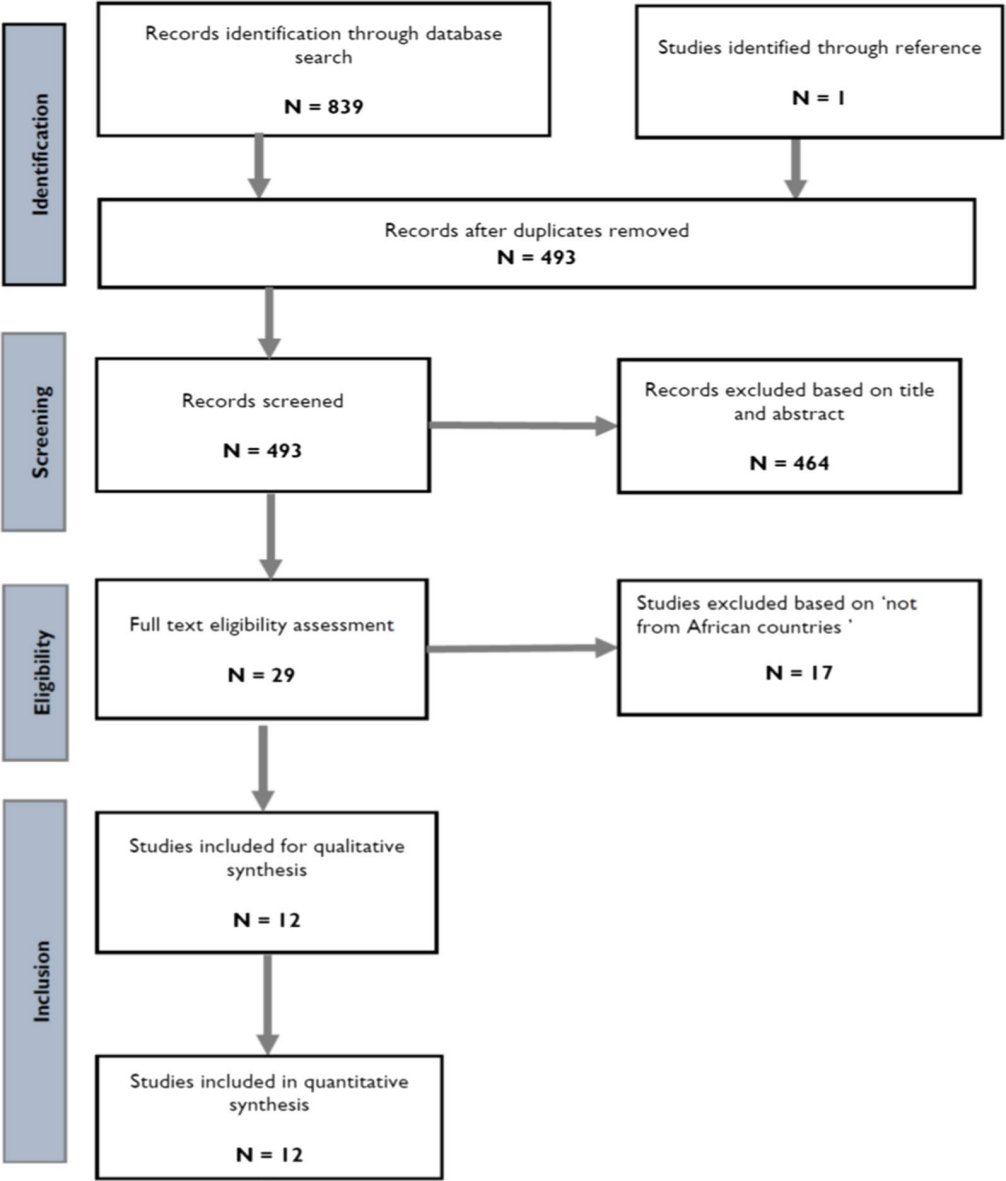
PRISMA flow diagram showing results of studies on yellow fever in Africa

We identified 839 studies from electronic search of five databases. After removing duplicates, we screened the titles and abstracts of 493 published articles and excluded 464 studies. We retrieved the full texts of the remaining 29 studies and excluded 12 of these studies [1, 11, 16–25] because they either did not report on the yellow fever burden or were from non-African countries. From the 17 studies that reported on yellow fever incidence, we excluded five more studies [18, 21–24] because they only reported on the evaluation of yellow fever vaccine without reporting data on yellow fever burden. We included a final total of 12 studies (Fig. 2) [2, 26–36].

### Characteristics of included studies

A summary table of characteristics of the included studies is presented in the appendices (Additional file 1: Table S2) We categorized studies based on year of assessment, test methods implemented for confirming the diagnosis of yellow fever, and clinical case definition used. The number of studies published each year increased modestly from 1975 to 2018. Although studies originated from eight African countries, three studies were from Nigeria [27–29], two studies each from Senegal [31, 32] and Uganda [33, 34] and one study from each of the five countries, Kenya [30], Ethiopia [34], Ghana [26], Gambia [2] and Democratic Republic of Congo [35]. The majority of studies ‘Seven’, were from West Africa [2, 26–32] ‘Three’ studies from East Africa [30, 33, 36], ‘One’ from North Eastern Africa [34] and ‘One’ study from Central Africa [35]. All the three modes of yellow fever transmission considered in this review were reported by at least one study. Eight studies reported that the outbreak was due to sylvatic mode of transmission [2, 27, 30–34, 36], two studies reported that it was due to an urban mode of transmission [28, 35] and only one study reported the intermediate mode of transmission [36].

Ten of the studies [2, 26–29, 31–36] were reported from hospital and field-based surveillance studies while two studies [30, 33] were only from hospital-based surveillance studies. Nine studies [2, 26–29, 31, 32, 36] included participants of all ages while four studies reported the disease in specific age groups such as from 10–70 years [30], 3 months–83 years [33], 3–64 years [36] and 10–72 years [35], respectively. Nine of the included studies [2, 26, 29–34, 36] were conducted in rural areas, two [28, 35] in urban areas and one [34] in mixed rural/urban setting. The population reported in all the studies lived on subsistence farming, growing several crops and rearing livestock. Most of the population under study practiced domestic water storage except for one study [34] that reported people practicing less livestock rearing and less domestic water storage while one study [35] did not report on any of the above. Rainy season and increased breeding sites were reported as risk factors for yellow fever epidemic as these can increase the mosquito population. However, two studies [31, 36] did not attribute the outbreak to the rains rather reported that the study population were in contact with the forest for 2 years before returning from the Internally Displaced Persons camps. They found their homes dirty exposing them to multiple natural breeding sites for mosquitoes that could have led to the outbreak. All the included studies also reported that yellow fever was confirmed using viral serology by doing enzyme-linked immunosorbent assay (ELISA) to detect IgM antibodies to yellow fever virus, and to isolate the virus. Some of the studies for instance [28, 30], reported that histopathology on liver specimens were done. One study, reported that test for antibody neutralization was not done [36].

The duration of the outbreak lasted for 11 months in Ethiopia [34] followed by Gambia which lasted for 8 months [2]. Outbreaks in Democratic Republic of Congo [35], Kenya [30], and Nigeria [27] lasted for 7, 6 and 5 months, respectively. Another outbreak in Nigeria [30] lasted for 4 months. Two studies from Senegal [31] lasted for 2 months and 1 month respectively [32] reported only 1 month. The other studies reported that the outbreak lasted for 3 months [28, 31, 32], while the study done in Ghana did not report the duration of the outbreak [26].

### Assessment of risk of bias of included studies

We evaluated all the studies using the Hoy’s risk of bias tool [14]. Our summary assessment shows that ten studies were low risk of bias (83%) [2, 27–34, 36] and 2 studies were moderate risk of bias (17%) (Additional file 1: Table S3).

### Incidence of yellow fever

**Fig. 3:**
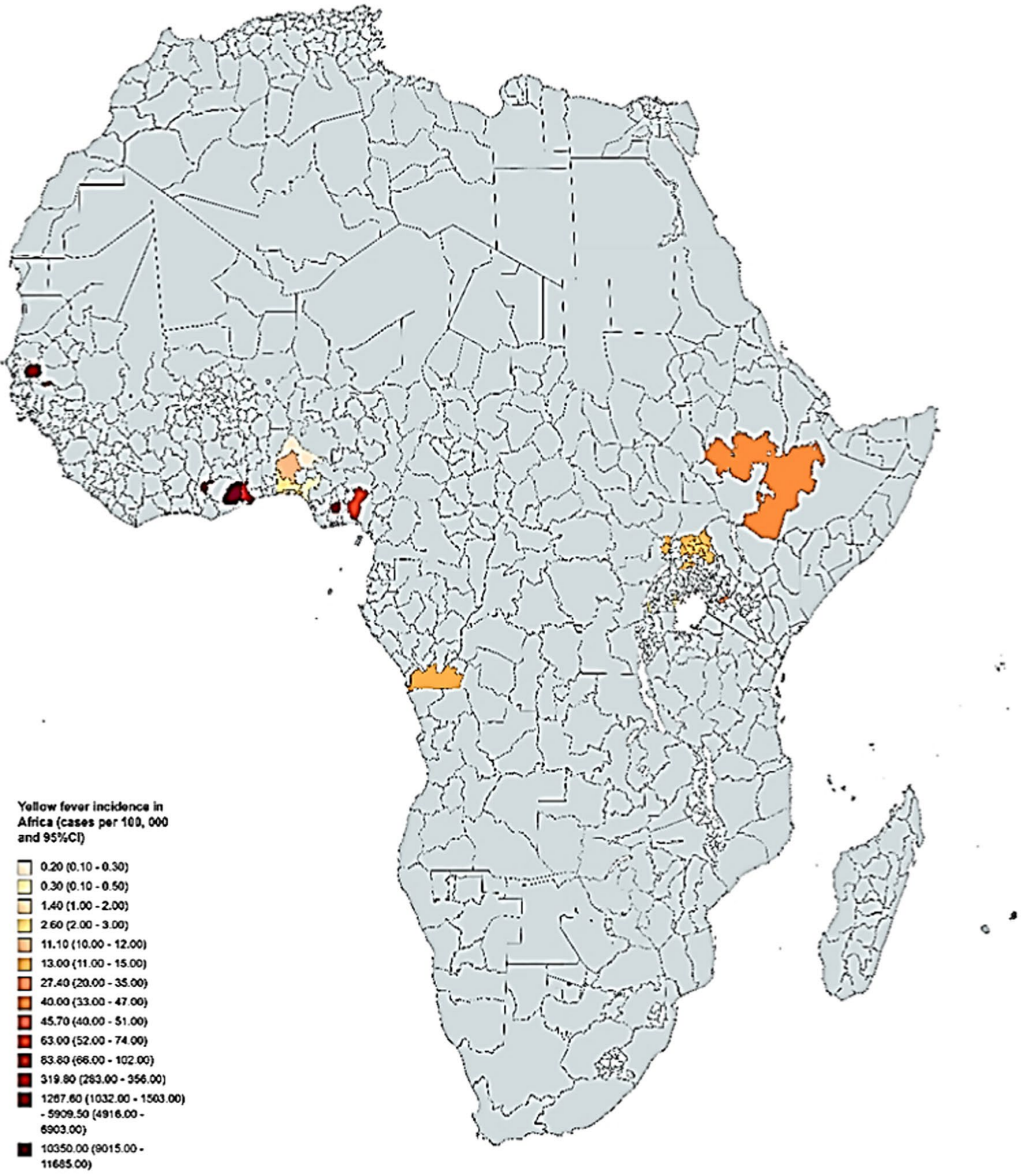
Map showing incidence rates (cases per 100,000) of yellow fever in Africa

Meta-analysis of yellow fever incidence estimates from different studies and countries resulted in significant heterogeneity (I^2^ = 99.4%, p < 0.001) and therefore we map the results per study/region in Africa (Fig. 3). The two studies from Uganda reported very low incidence of less than 3 and 13 cases per 100,000 population respectively, Kenya < 30 cases per 100,000, Ethiopian 40 cases per 100,000, In Gambia < 50 cases per 100,000, Nigeria the incidence ranged from < 1 to over 80 cases per 100,000 population. The two studies in Senegal reported incidence rates of approximately 1300 and 5900 cases per 100,000 population while Ghana reported the highest incidence which ranged between 320 to over 10,000 cases per 100,000 population (Fig. 3).

### Case fatality rate

**Fig. 4:**
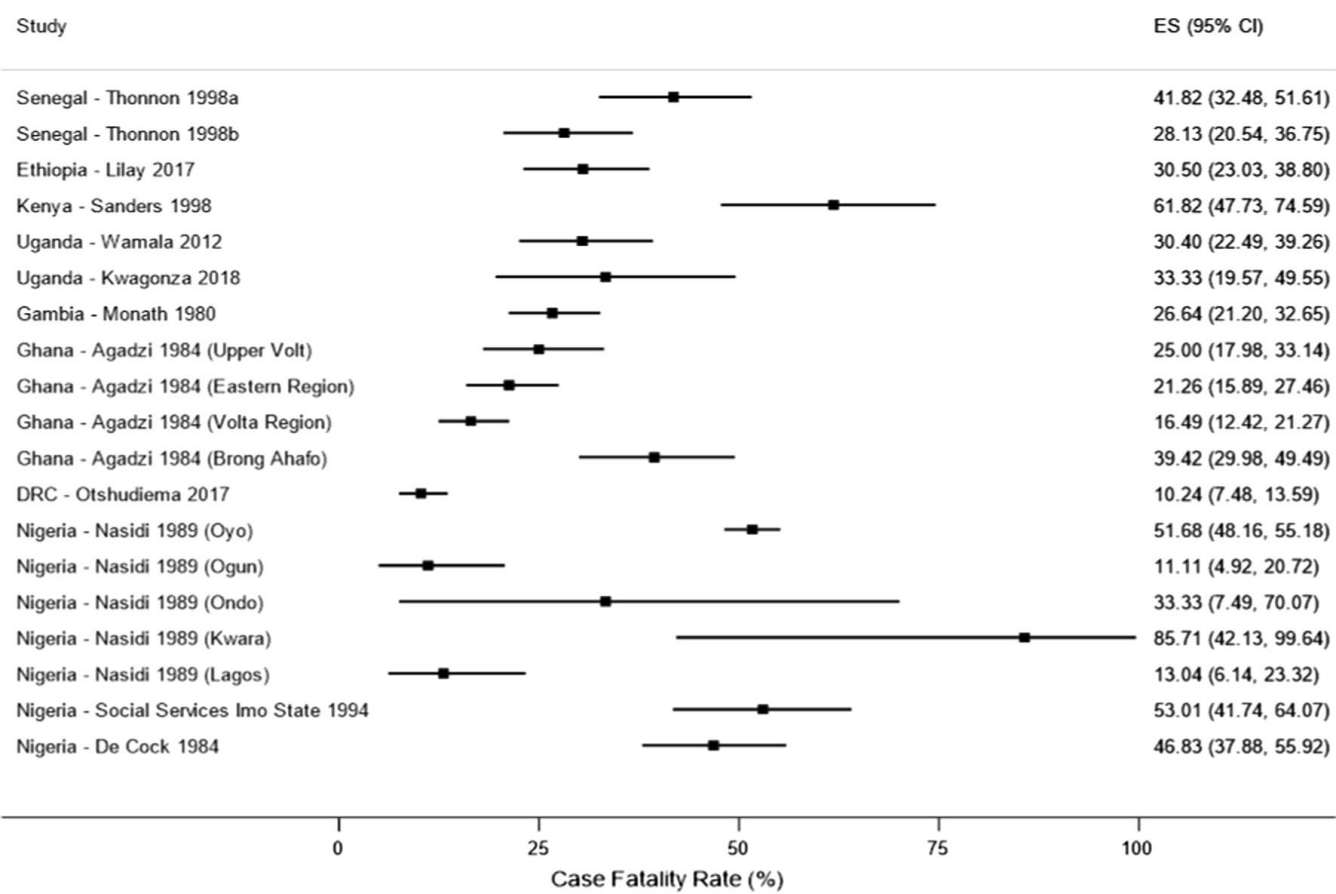
Forest plot showing case fatality rates associated with yellow fever outbreaks in Africa

Meta-analysis of CFR (in %) resulted in significant heterogeneity (I^2^ = 95.6%, p < 0.001, Fig. 3) and therefore we report results narratively per study. The only study from Democratic Republic of Congo had the lowest CFR of just over 10%. The Ethiopian study reported a CFR of slightly over 30%, the two studies from Uganda reported CFRs of just over 30% each, while the one study from Gambia reported a CFR of less than 30%. The CFRs in different regions of Ghana ranged from just over 16% in Volta Region to almost 40% in Brong Ahafo. The two Senegal studies reported high CFRs of 28% and 42%, while the Kenyan study reported a higher CFR of over 60%. Lastly, the Nigerian studies reported varying CFRs from 11 to 85% (Fig. 4).

### Mortality rate

We could not perform meta-analysis of mortality rate because the population sizes were not reported by most included studies. The results also differed widely such that a meta-analysis would likely result in high heterogeneity. We therefore reported the results narratively for each study. The two studies from Senegal had high mortality rates ranging from over 500 to almost 1700 deaths per 100,000. The Ethiopian and Kenyan single studies had low mortality rates of 12 and 17 deaths per 100,000 population. In Uganda, the mortality rates were even lower, ranging from less than 1 to 4 deaths per 100,000 population. In Gambia, it was 12 deaths per 100,000 population. In Ghana, the mortality rate ranged from just over 50 in Volta Region to over 2200 deaths per 100,000 population in the Eastern Region. The one study in DRC reported a low mortality rate of 1 death per 100,000 population. Lastly, the studies from Nigeria reported mortality rates ranging from 0.2 deaths per 100,000 population in Kwara State to more than 44 deaths per 100,000 population in South Eastern Nigeria.

## Discussion

We carried out this systematic review with the objective of estimating the incidence and associated mortality of yellow fever in Africa. We found 12 studies from eight countries in Africa, suggesting that studies are scarce in yellow fever-endemic African countries. We found in studies in Nigeria [27–29], Uganda [33, 36], Senegal [31, 32] Kenya [30], Ethiopia [34], Gambia [2], Ghana [26] and Democratic Republic of Congo [35]. ‘Eight’ studies were undertaken more than 20 years ago, and with only four recent studies. This shows there is scarcity of data, thereby making the true estimation of yellow fever incidence difficult in Africa. However, data reported that yellow fever is a disease of the East and West African countries. We found that incidence of yellow fever varies in different countries. The incidence of yellow fever was highest in Senegal [31, 32] (1300–5900/100,000 followed by 320/100,000 in Ghana [26] and lowest in Uganda with a range of 3–13/100,000 [33, 36]. The reason for this increase could be due to an improved surveillance reporting system. Case fatality rate was highest (60%) in Kenya and ranged from 11 to 85% in Nigeria while Democratic Republic of Congo had the lowest case fatality rate of over 10% [28, 30, 35]. Finally, mortality rate was highest in Senegal [31, 32] 500–1700 deaths/100,000, Uganda [33, 36] had a low mortality rate of less than 1 to 4 deaths/100,000 while Nigeria ranged from 0.2 to 44 deaths/100,000 [27–29]. These results illustrated that the case fatality and mortality rates widely vary in the African yellow fever belt. In comparison, a 25-year study in Africa estimated that 13% (95% CI 5–28%) of yellow fever infections presented as severe cases, with 46% (95% CI 31–60%) of severe cases resulting in death [37]. These findings could also be as a result of better surveillance system and global awareness now compared to what we had previously. Some studies report that a combination of some factors like socioeconomic, demographic and ecological circumstances favoured the high incidence of yellow fever in Africa [36]. Other authors have indicated that Africa monkey are resistant to the yellow fever virus and if they become infected they usually do not die but rather become immune and humans become accidentally infected during their short forest activities [36]. Usually African population are at a higher risk of the disease because of very low immunity. This is due to low vaccination coverage and also linked to their daily activities and occupation such as farming, deforestation, and cattle grazing, which bring them in close proximity to the forest where the vectors live.

From the studies that we included in this review, the outbreaks that occurred were due to sylvatic mode of transmission which involves the virus passed on between non-human primates (e.g., monkeys) and mosquito species found in the forest canopy [26–29, 32–36]. Yellow fever is endemic in the sylvatic settings in Africa predominantly in East and West Africa. The sylvatic mode of transmission gives a more regular epidemic pattern as opposed to urban transmission which often gives a periodic and more unpredictable outbreak. This information is ideal as it would help policy makers focus the strategies on how best to fight the regular clustering of yellow fever epidemic. Some of these strategies would include using an integrated mosquito management (IMM) [38] program steps such as: (i) surveillance: this will help detect the different mosquito species in a given area and with this data, they are able to effectively time larvicide and adulticide activities; (ii) public education: this includes educating the general public on how to control mosquito breeding sites at their backyard; (iii) larval and adult mosquito control: inspecting sources of standing water looking for mosquito larval so as to eliminate them before they become adults that can transmit the virus while the adult mosquitoes are treated using pesticides. We also noticed that yellow fever is endemic with the outbreaks occurring during rainy season. It is known that rainy season increases the breeding sites and the mosquito population which transmit the disease. Hence heavy and prolong rainfall result in high vector population due a favorable breeding environment for mosquitoes due to heavy rainfall during rainy season. This provides adequate ground to support continued circulation of the virus especially in the sylvatic cycle.

Previously, prevention of this disease was done through vector control which was very effective in reducing the occurrence of the disease. However, smaller outbreaks occur due to changes in the distribution of the disease and currently, vaccination strategies such as routine infant immunization, mass vaccination campaigns and vaccination of travelers going to yellow fever endemic regions are used to protect people against these outbreaks. Yellow fever vaccine called 17D is known for preventing the disease and just a single dose of the vaccine confers immunity and lifelong protection. The vaccine provides effective immunity within 10 days for 80–100% of people vaccinated, and within 30 days for more than 99% of people vaccinated [39]. Most African countries where yellow fever is endemic have included yellow fever vaccine in the Expanded Programme on Immunization (EPI) schedule for new-born babies. Most of the yellow fever cases identified in Africa are seen in the unvaccinated population who live in the yellow fever belt. Some countries have sustained epidemics across multiple years like Ghana (1977–83), Guinea (2000–2005), Nigeria (1986–1994) and Congo (2011–2013) [37]. Looking at previous data on yellow fever over a period of 25 years in Africa, showed that yellow fever has been persistent in West and Central Africa [37]. In East Africa, yellow fever has mostly been in Kenya [37]. However, cases were also reported in Uganda and Ethiopia. The estimated annual provincial incidence in 32 African countries known to be endemic for yellow fever varied from 0.7 to 10% [37] which is low compared to the overall incidence rates found by this systematic review. A recent study reported that in 2018 there were approximately 109,000 (95% CI 67,000–173,000) severe infections and 51,000 (95% CI 31,000–82,000) deaths due attributed to yellow fever in Democratic Republic of Congo and Amazon region [40].

In December 2016, cases of yellow fever were detected in Luanda, the capital of Angola, which were previously in the category of low-risk areas for yellow fever. The disease spread rapidly from Luanda to other urban communities in Angola and crossed into the neighbouring country of the Democratic Republic of Congo [16, 41]. This observation empathizes the fact that yellow fever is a neglected tropical disease which needs more public health strategies to contain it coupled with more studies to be conducted in Africa to generate in-depth evidence for effective interventions.

Reviewing the vaccination coverage, WHO recommends population vaccination coverage of 80% or more to prevent and control outbreaks. However, all of the regions included in this review has recorded low vaccination coverage. A recent study showed the targeted versus the untargeted vaccination coverage of 54%/63% and 24%/24% in Democratic Republic of the Congo (Central Kongo region), 61%/75% in Senegal (Kaffrine region), zero coverage in Ethiopia (South Omo zone), 67%/67% in Ghana (Volta, Brong Ahafo and Eastern Regions), zero coverage in Kenya (Kerio Valley), zero coverage in Uganda (Northern Uganda, Masaka, Kalungu, Kalangala and Rukungire regions) and 56%/65% in Nigeria (Cross river, Lagos, Oyo, Ogun, Ondo, Kwara and Imo state) [42]. As shown, all of the regions with a high incidence of yellow fever and case fatality rate in this review had less than 80% vaccination coverage, with some regions having 0%.

The review has shown that yellow fever incidence seems to have been pretty constant throughout African countries over the inclusion period of the review. However, three of the studies included showed high incidence rates [26, 31, 32]. In contrast, the fatality rates varied widely across African countries over the same period of the review. Since most of the studies were conducted between 1984 and 1998, moderately high vaccination coverage rates across much of western and central Africa in the 1970s were the result of mass preventive campaigns in the 1940s to the 1960s [42, 43], which reduced the number of outbreaks [43]. Coverage declined between 1960 and 2000 in most areas due to limited vaccination activity, the birth of new unvaccinated cohorts, a steady decline in the proportion of older covered cohorts through mortality [43] and vaccine stock shortages, which have been frequently reported in the African region [44]. Past public health successes led to a lax in maintaining local yellow fever vaccination coverage leading to waning herd immunity and an eventual re-emergence of large outbreaks in West Africa in the 2000s [42, 45].

In fact, yellow fever may not easily be eliminated due to the presence of non-human wildlife reservoirs that sustain the sylvatic transmission cycle of the virus in non-urban settings, however the risk of a yellow fever outbreak can be eliminated if successful vector control, vaccination and surveillance of the disease are implemented and maintained [42]. The joint effort by the WHO, the United Nations International Children's Emergency Fund (UNICEF), the Global Alliance for Vaccinations and Immunization (GAVI) and yellow fever virus (YFV) endemic countries created the Yellow Fever Initiative (YFI) in 2006 [46], which focused primarily on widespread yellow fever vaccination initiatives and the implementation of childhood immunization vaccine. In the context of an emergency preparedness effort, this initiative has created an opportunity for global stock of yellow fever vaccines [41, 47]. The 17D yellow fever vaccine is effective, safe, affordable, readily available, and can prevent the disease with just a single dose being sufficient enough to confer sustained immunity and lifelong protection.

The review has limitations the main one being paucity of yellow fever incidence/prevalence data from different African countries. The incidence estimates from the different studies were significantly heterogeneous and there were not enough data from the studies to determine the sources of this heterogeneity, but could have been due to the different geographical nature in Africa, seasons, settings (rural or urban), different populations, statistical methods and regional differences. Studies included in this review were fewer hence limiting the scope to generate more accurate estimates of the burden of yellow fever in African regions. Secondly most of the included studies were predominantly from East–West Africa, hence understanding the burden of yellow fever in other regions is restricted.

The risk of bias assessment showed that 83% of studies showed to have a low risk of bias, however included studies mighty not have been representative of a larger population. Furthermore, in most of the included studies, the number of confirmed and suspected cases were not clearly distinguished. As such this limits generalizability of the incidence and case fatality rate estimates to a larger African population.

## Conclusion

This systematic review identified 12 observational studies assessing yellow fever incidence and fatality rates in Africa. Data shows that yellow fever incidence rate is quite constant across African countries with high incidence rates reported in three studies of the 12 studies. The case fatality rates for yellow fever varied widely across Africa. However, the lack of reliable epidemiological data on yellow fever in Africa compromises the public health priority that could give rise to yellow fever infection.

For that reason, it is essential to provide standardized demographic health surveys as a population surveillance strategy to track the burden of yellow fever as well as accurate diagnostic measures for early recognition and treatment of yellow fever. Knowing that yellow fever usually re-emerges in rural areas, rural facilities should be enforced in yellow fever prevention, management and reporting approach. Timely and accurate diagnosis of yellow fever could avoid untoward case fatality rates and minimize under-reporting. Accurate yellow fever data is substantial for good public health policy and guide planning of vaccine volumes and delivery system. In addition, public health control strategy should focus on strengthening yellow fever prevention including incorporating yellow fever immunization schedules in African endemic countries and mandatory reporting of cases in primary, secondary and tertiary levels. Future research should focus on evaluating yellow fever immunogenicity in children.

## Supplementary Information

**Additional file 1: Supplementary Table S1.** Included the search strategy: MEDLINE (PubMed), SCOPUS, Africa-wide (EBSCOhost), Web of science (SCI-EXPANDED), CINAHL and Embase. **Supplementary Table S2.** Characteristics of included studies of yellow fever in Africa. **Supplementary Table S3.** Risk of bias assessment of included studies (Adapted from Hoy et al, 2012).

## Data Availability

All data generated or analysed during this study are included in this published article (Supplementary table 2).

## Abbreviations

*Ae*: *Aedes*
CFR: Case fatality rate
ELISA: Enzyme-linked immunosorbent assay
EPI: Expanded Programme on Immunization
ICID: International Congress on Infectious Diseases
ICIDD: International Conference on Infectious Disease Dynamics
IMM: Integrated mosquito management
ISID: International Society for Infectious Diseases
GAVI: Global Alliance for Vaccinations and Immunization
IgG: Immunoglobulin G
IgM: Immunoglobulin M
MeSH: Medical Subject Heading
UNICEF: United Nations International Children’s Emergency Fund
WHO: World Health Organization
YFI: Yellow Fever Initiative
YFV: Yellow fever virus

## References

[1] Kwallah AO, Inoue S, Thairu-Muigai AW (2014). Sero-prevalence of yellow fever virus in selected health facilities in Western Kenya from 2010 to 2012. Jpn J Infect Dis.

[2] Monath TP, Craven RB, Adjukiewicz A (1980). Yellow fever in the Gambia, 1978–1979: epidemiologic aspects with observations on the occurrence of orungo virus infections. Am J Trop Med Hyg.

[3] CDC (2021). Yellow fever vaccine recommendations.

[4] CDC (2019). Transmission of yellow fever virus.

[5] WHO (2014). Yellow fever. Rapid field entomological assessment during yellow fever outbreaks in Africa.

[6] WHO (2016). Fractional dose yellow fever vaccine as a dose-sparing option for outbreak response: WHO Secretariat information paper (No. WHO/YF/SAGE/16.1).

[7] Mutebi JP, Barrett AD (2002). The epidemiology of yellow fever in Africa. Microbes Infect.

[8] Reliefweb. Fight against yellow fever: Institut Pasteur Foundation in Dakar to set up new vaccine production unit. 2016.

[9] WHO (2010). Yellow fever initiative. Providing an opportunity of a lifetime.

[10] WHO (2016). Situation report: yellow fever outbreak in Angola.

[11] Gubler DJ (2002). The global emergence/resurgence of arboviral diseases as public health problems. Arch Med Res.

[12] Marlow MA, de Feliciano Pambasange MAC, Francisco C (2017). Notes from the field: knowledge, attitudes, and practices regarding yellow fever vaccination among men during an outbreak—Luanda, Angola, 2016. MMWR Morb Mortal Wkly Rep.

[13] WHO (2019). Yellow fever.

[14] Hoy D, Brooks P, Woolf A (2012). Assessing risk of bias in prevalence studies: modification of an existing tool and evidence of interracter agreement. J Clin Epidemiol.

[15] Schroll JB, Moustgaard R, Gøtzsche PC (2011). Dealing with substantial heterogeneity in Cochrane reviews. Cross-sectional study. BMC Med Res Methodol.

[16] Kraemer MU, Faria NR, Reiner JRC (2017). Spread of yellow fever virus outbreak in Angola and the Democratic Republic of the Congo 2015–16: a modelling study. Lancet Infect Dis.

[17] Tsai TF, Lazuick JS, Ngah RW (1987). Investigation of a possible yellow fever epidemic and serosurvey for flavivirus infections in northern Cameroon, 1984. Bull World Health Organ.

[18] Onyango CO, Grobbelaar AA, Gibson GVF (2004). Yellow fever outbreak, Southern Sudan, 2003. Emerg Infect Dis.

[19] Ellis BR, Barrett AD (2008). The enigma of yellow fever in East Africa. Rev Med Virol.

[20] Wiysonge CS, Nomo E, Mawo J (2008). Yellow fever control in Cameroon: where are we now and where are we going?. BMC Med.

[21] Farnon EC, Gould LH, Griffith KS (2010). Household-based sero-epidemiologic survey after a yellow fever epidemic, Sudan, 2005. Am J Trop Med Hyg.

[22] Attoh-Touré H, Dagnan NS, Tagliante-Saracino J (2010). Resurgence of yellow fever epidemics in Côte-d'Ivoire. Bulletin de la Société de Pathologie Exotique.

[23] Green A (2015). Yellow fever continues to spread in Angola Health workers in Angola warn that although the number of newly confirmed cases of yellow. Lancet.

[24] Grobbelaar AA, Weyer J, Moolla N (2016). Resurgence of yellow fever in Angola, 2015–2016. Emerg Infect Dis.

[25] Nishino K, Yactayo S, Garcia E (2016). Yellow fever urban outbreak in Angola and the risk of extension Flambée urbaine de fièvre jaune en Angola et risque d’ extension. Wkly Epidemiol Rec.

[26] Agadzi VK, Boatin BA, Appawu MA (1984). Yellow fever in Ghana, 1977–80. Bull World Health Organ.

[27] De Cock KM, Nasidi A, Enriquez J (1988). Epidemic yellow fever in eastern Nigeria, 1986. The Lancet.

[28] Nasidi A, Monath TP, DeCock K (1989). Urban yellow fever epidemic in western Nigeria, 1987. Trans R Soc Trop Med Hyg.

[29] WHO (1995). Yellow fever: investigation of an epidemic in Imo State. Wkly Epidemiol Rec.

[30] Sanders EJ, Marfin AA, Tukei PM (1998). First recorded outbreak of yellow fever in Kenya, 1992–1993. I. Epidemiologic investigations. Am J Trop Med Hyg.

[31] Thonnon J, Fontenille D, Tall A (1998). Re-emergence of yellow fever in Senegal in 1995. Am J Trop Med Hyg.

[32] Thonnon J, Spiegel A, Diallo M (1998). Yellow fever outbreak in Kaffrine, Senegal, 1996: epidemiological and entomological findings. Trop Med Int Health.

[33] Wamala JF, Malimbo M, Okot CL (2012). Epidemiological and laboratory characterization of a yellow fever outbreak in northern Uganda, October 2010–January 2011. Int J Infect Dis.

[34] Lilay A, Asamene N, Bekele A (2017). Reemergence of yellow fever in Ethiopia after 50 years, 2013: epidemiological and entomological investigations. BMC Infect Dis.

[35] Otshudiema JO, Ndakala NG, Mawanda EK (2017). Yellow fever outbreak-Kongo Central Province, Democratic Republic of the Congo, August 2016. MMWR Morb Mortal Wkly Rep.

[36] Kwagonza L, Masiira B, Kyobe-bosa H (2018). Outbreak of yellow fever in central and southwestern Uganda, February–May 2016. BMC Infect Dis.

[37] Garske T, Van Kerkhove MD, Yactayo S, Ronveaux O, Lewis RF (2014). Yellow fever in Africa: estimating the burden of disease and impact of mass vaccination from outbreak and serological data. PLoS Med.

[38] WHO (2008). Detection and investigation of serious adverse events following yellow fever vaccination, Guidance from an informal consultation of experts, 18–19 November 2008.

[39] Baba MM, Ikusemoran M (2017). Is the absence or intermittent YF vaccination the major contributor to its persistent outbreaks in eastern Africa?. Biochem Biophys Res Commun.

[40] Gaythorpe KAM, Hamlet ATP, Jean K (2020). The global burden of yellow fever. Medxrix.

[41] Bifani AM, Ong EZ, de Alwis R (2020). Vaccination and therapeutics: responding to the changing epidemiology of yellow fever. Curr Treat Options Infect Dis.

[42] Shearer FM, Moyes CL, Pigott DM (2017). Global yellow fever vaccination coverage from 1970 to 2016: an adjusted retrospective analysis. Lancet Infect Dis.

[43] Durieux C. Mass yellow fever vaccination in French Africa south of the Sahara. Yellow Fever Vaccination, Monograph Series. 1956;30:115–21.

[44] WHO (2018). Eliminate yellow fever epidemics (EYE): a global strategy, 2017–2026.

[45] Rogers DJ, Wilson AJ, Hay SI, Graham AJ (2006). The global distribution of yellow fever and dengue. Adv Parasitol.

[46] WHO (2010). Yellow fever initiative: providing an opportunity of a lifetime.

[47] WHO (2020). International coordination group on vaccine provision for yellow fever: report of the annual meeting.

