# Supplementary material for: The incidence and mortality of yellow fever in Africa: a systematic review and meta-analysis
Source: BMC Infect Dis. 2021 Oct 23;21:1089. doi: 10.1186/s12879-021-06728-x (PMC8536483; doi:10.1186/s12879-021-06728-x)
Supplement: Supplementary file 1 — Additional file 1: Supplementary Table S1. Included the search strategy: MEDLINE (PubMed), SCOPUS, Africa-wide (EBSCOhost), Web of science (SCI-EXPANDED), CINAHL and Embase. Supplementary Table S2. Characteristics of included studies of yellow fever in Africa. Supplementary Table S3. Risk of bias assessment of included studies (Adapted from Hoy et al, 2012). [file 12879_2021_6728_MOESM1_ESM.docx]

**Additional files**

**search strategy 1: MEDLINE (PubMed)**

1 Developing Countries OR Africa OR Africa, Northern OR Africa South of the Sahara OR Africa, Central OR Africa, Eastern OR Africa, Southern OR Africa, Western OR Angola OR Benin OR Botswana OR Burkina Faso OR Burundi OR Cameroon OR Cape Verde OR Central African Republic OR Chad OR Comoros OR Congo OR Cote d'Ivoire OR Djibouti OR "Democratic Republic of the Congo" OR Eritrea OR Ethiopia OR Gabon OR Gambia OR Ghana OR Guinea OR Guinea-Bissau OR Kenya OR Lesotho OR Liberia OR Libya OR Madagascar OR Malawi OR Mali OR Mauritania OR Mauritius OR Mozambique OR Namibia OR Niger OR Nigeria OR Rwanda OR Senegal OR Seychelles OR Sierra Leone OR Somalia OR South Africa OR Sudan OR Swaziland OR Tanzania OR Togo OR Uganda OR Zambia OR Zimbabwe

2 Madagascar OR Malawi OR Mali OR Mauritania OR Mauritius OR Mozambique OR Namibia OR Niger OR Nigeria OR Rwanda OR Sao Tome OR Senegal OR Seychelles OR Sierra Leone OR Somalia OR Sudan OR Swaziland OR Tanzania OR Togo OR Uganda OR Zambia OR Zimbabwe OR Rhodesia

3 Africa OR Angola OR Benin OR Botswana OR Burkina Faso OR Burkina Faso OR Upper Volta OR Burundi OR Cameroon OR Cape Verde OR Central African Republic OR Chad OR Comoros OR Comoro Islands OR Comores OR Mayotte OR Congo OR Zaire OR Cote d'Ivoire OR Ivory Coast OR Djibouti OR Somalia OR Ethiopia OR Gabon OR Gabonese Republic OR Gambia OR Ghana OR Guinea OR Guiana OR Kenya OR Lesotho OR Liberia OR Libya

4 "developing country" OR "developing countries" OR "developing nation" OR "developing nations" OR "developing population" OR "developing populations" OR "developing world" OR "less developed country" OR "less developed countries" OR "less developed nation" OR "less developed nations" OR "less developed population" OR "less developed populations" OR "less developed world" OR "lesser developed country" OR "lesser developed countries" OR "lesser developed nation" OR "lesser developed nations" OR "lesser developed population" OR "lesser developed populations" OR "lesser developed world" OR "under developed country" OR "under developed countries" OR "under developed nation” OR "under developed nations" OR "under developed population" OR "under developed populations" OR "under developed world" OR "underdeveloped country" OR "underdeveloped countries" OR "underdeveloped nation" OR "underdeveloped nations" OR "underdeveloped population" OR "underdeveloped populations" OR "underdeveloped world" OR "middle income country" OR "middle income countries" OR "middle income natiu1on" OR "middle income nations" OR "middle income population" OR "middle income populations" OR "low income country" OR "low income countries" OR "low income nation" OR "low income nations" OR "low income population" OR "low income populations” OR "lower income country “OR "lower income countries" OR "lower income nation" OR "lower income nations “OR "lower income population" OR "lower income populations" OR "underserved country" OR "underserved countries" OR "underserved nation" OR "underserved nations" OR "underserved population" OR "underserved populations" OR "underserved world" OR "underserved country" OR "underserved countries" OR "underserved nation" OR "underserved nations" OR "underserved population" OR "underserved populations “OR "underserved world" OR "deprived country" OR "deprived countries" OR "deprived nation “OR "deprived nations" OR "deprived population" OR "deprived populations" OR "deprived world" OR "poor country" OR "poor countries" OR "poor nation" OR "poor nations" OR "poor population" OR "poor populations" OR "poor world" OR "poorer country" OR "poorer countries" OR "poorer nation" OR "poorer nations" OR "poorer population" OR "poorer populations" OR "poorer world" OR "developing economy" OR "developing economies" OR "less developed economy" OR "less developed economies" OR "lesser developed economy" OR "lesser developed economies" OR "under developed economy" OR "under developed economies" OR "underdeveloped economy" OR “underdeveloped economies" OR "middle income economy" OR "middle income economies" OR "low income economy" OR "low income economies" OR "lower income economy" OR "lower income economies" OR "low gdp" OR "low gnp" OR "low gross domestic" OR "low gross national" OR "lower gdp" OR "lower gnp" OR "lower gross domestic" OR "lower gross national" OR lmic OR “lmics" OR "third world" OR "lami country" OR "lami countries" OR "transitional country" OR "transitional countries"

5 1 0R 2 OR 3 OR 4

6 “yellow fever” OR “yellow jack”

7 prevalence OR "Prevalence"

8 5 AND 6 AND 7

**Search strategy 2: Scopus**

( ( ( TITLE-ABS-KEY ((((Developing Countries OR Africa OR Africa, Northern OR Africa South of the Sahara OR Africa, Central OR Africa, Eastern OR Africa, Southern OR Africa, Western OR Angola OR Benin OR Botswana OR Burkina Faso OR Burundi OR Cameroon OR Cape Verde OR Central African Republic OR Chad OR Comoros OR Congo OR Cote d'Ivoire OR Djibouti OR "Democratic Republic of the Congo" OR Eritrea OR Ethiopia OR Gabon OR Gambia OR Ghana OR Guinea OR Guinea-Bissau OR Kenya OR Lesotho OR Liberia OR Libya OR Madagascar OR Malawi OR Mali OR Mauritania OR Mauritius OR Mozambique OR Namibia OR Niger OR Nigeria OR Rwanda OR Senegal OR Seychelles OR Sierra Leone OR Somalia OR South Africa OR Sudan OR Swaziland OR Tanzania OR Togo OR Uganda OR Zambia OR Zimbabwe) ) ) ) )

AND

((TITLE-ABS-KEY (yellow fever) OR TITLE-ABS-KEY (yellow jack) OR TITLEABS-KEY (yellow fevers) OR (haemorrhagic fever)

AND

(“prevalence” OR "Prevalence")

AND

(“outbreak” OR “Burden of disease” OR “Incidence”)

**Search strategy 3: Web of Science**

(((Developing Countries OR Africa OR Africa, Northern OR Africa South of the Sahara OR Africa, Central OR Africa, Eastern OR Africa, Southern OR Africa, Western OR Angola OR Benin OR Botswana OR Burkina Faso OR Burundi OR Cameroon OR Cape Verde OR Central African Republic OR Chad OR Comoros OR Congo OR Cote d'Ivoire OR Djibouti OR "Democratic Republic of the Congo" OR Eritrea OR Ethiopia OR Gabon OR Gambia OR Ghana OR Guinea OR Guinea-Bissau OR Kenya OR Lesotho OR Liberia OR Libya OR Madagascar OR Malawi OR Mali OR Mauritania OR Mauritius OR Mozambique OR Namibia OR Niger OR Nigeria OR Rwanda OR Senegal OR Seychelles OR Sierra Leone OR Somalia OR South Africa OR Sudan OR Swaziland OR Tanzania OR Togo OR Uganda OR Zambia OR Zimbabwe)))) AND

TOPIC: ((“yellow fever” OR “yellow jack” OR “yellow fevers”)) AND

TOPIC: ((“prevalence” OR "Prevalence")) AND

TOPIC: ((outbreak OR “Burden of disease” OR “Incidence”)

**Search strategy 4: EBSCOhost**

S1

Developing Countries OR Africa OR Africa, Northern OR Africa South of the Sahara OR Africa, Central OR Africa, Eastern OR Africa, Southern OR Africa, Western OR Angola OR Benin OR Botswana OR Burkina Faso OR Burundi OR Cameroon OR Cape Verde OR Central African Republic OR Chad OR Comoros OR Congo OR Cote d'Ivoire OR Djibouti OR "Democratic Republic of the Congo" OR Eritrea OR Ethiopia OR Gabon OR Gambia OR Ghana OR Guinea OR Guinea-Bissau OR Kenya OR Lesotho OR Liberia OR Libya OR Madagascar OR Malawi OR Mali OR Mauritania OR Mauritius OR Mozambique OR Namibia OR Niger OR Nigeria OR Rwanda OR Senegal OR Seychelles OR Sierra Leone OR Somalia OR South Africa OR Sudan OR Swaziland OR Tanzania OR Togo OR Uganda OR Zambia OR Zimbabwe

S2

Madagascar OR Malawi OR Mali OR Mauritania OR Mauritius OR Mozambique OR Namibia OR Niger OR Nigeria OR Rwanda OR Sao Tome OR Senegal OR Seychelles OR Sierra Leone OR Somalia OR Sudan OR Swaziland OR Tanzania OR Togo OR Uganda OR Zambia OR Zimbabwe OR Rhodesia

S3

Africa OR Angola OR Benin OR Botswana OR Burkina Faso OR Burkina Faso OR Upper Volta OR Burundi OR Cameroon OR Cape Verde OR Central African Republic OR Chad OR Comoros OR Comoro Islands OR Comores OR Mayotte OR Congo OR Zaire OR Cote d'Ivoire OR Ivory Coast OR Djibouti OR Somalia OR Ethiopia OR Gabon OR Gabonese Republic OR Gambia OR Ghana OR Guinea OR Guiana OR Kenya OR Lesotho OR Liberia OR Libya

S4

TI (low 3 middle N3 countr*) OR AB (low 3 middle N3 countr*) OR SU (low 3 middle N3 countr*)

S5

TI ((developing or less* N1 developed or "under developed" or underdeveloped or "middle income" or low* N1 income) N1 (economy or economies)) OR AB ((developing or less* N1 developed or "under developed" or underdeveloped or "middle income" or low* N1 income) N1 (economy or economies)) OR SU ((developing or less* N1 developed or "under developed" or underdeveloped or "middle income" or low* N1 income) N1 (economy or economies))

S6

yellow fever OR yellow jack OR yellow fevers

S7

prevalence OR Prevalence

S8

“outbreak” OR “Burden of disease” OR “Incidence”

**Search strategy 5: Embase and CINAHL**

Controlled vocabulary (subject headings)

**Table S2: Characteristics of included studies of yellow fever in Africa**

| **Study ID/Region** | **Year of assessment (duration)** | **Country** | **Mode of transmission** | **Population** | **Study design/type** | **Outcomes** | **Hospital vs field based** | **Rural / urban/mixed** |
| --- | --- | --- | --- | --- | --- | --- | --- | --- |
| Thonnon 1998a//Ribo-Escale and Guente-Pate | 1995(1 month) | Senegal | Intermediate | Cases: 110  All ages | Case detection and sero-survey | Death: 46  Attack rate: 0,0126  Incidence per 100000: 1267,6  Case fatality rate (%): 41.8  Mortality per 100000: 530,1 | Hospital & field | Rural |
| Thonnon 1998b/ Kounghoul & Affrine | 1995(8 days) | Senegal | Sylvatic | Cases: 128  All ages | Case finding & surveillance | Death: 36  Attack rate: 0,059095106  Incidence per 100000: 5909,5  Case fatality rate (%):28,1  Mortality per 100000: 1662 | Hospital & field | Rural |
| Lilay et al. 2017/ South Omo zone | 2013(2months) | Ethiopia | Sylvatic | Cases: 141  All ages | Case finding & survey | Death: 43  Attack rate: 0,0004  Incidence per 100000: 40  Case fatality rate (%): 30,5  Mortality per 100000: 12.2 | Hospital & field | Mixed |
| Sanders et al. 1998/ Kerio Valley | 19936months) | Kenya | Sylvatic | Cases: 55  10 – 70 years | Retrospective and prospective case surveillance | Death: 34  Attack rate: 0,000274  Incidence per 100000: 27,4  Case fatality rate (%): 61,8  Mortality per 100000: 16.9 | Hospital | Rural |
| Wamala et al. 2012/ Northen Uganda | 2010 (1month) | Uganda | Sylvatic | Cases: 125  3monts -83yrs | Case series | Death: 38  Attack rate: 0,00013  Incidence per 100000: 13  Case fatality rate (%): 30.4  Mortality per 100000: 4 | Hospital & field | Rural |
| Kwagonza et al. 2018/ Masaka, Kalungu, Kalangala & Rukungire | 2018(3months) | Uganda | Sylvatic | Cases: 42  3 – 64 years | Case control | Death: 14  Attack rate: 0,000026  Incidence per  100000: 2.6  Case fatality rate (%): 33.3  Mortality per 100000: 0.9 | Hospital & field | Rural |
| Monath et al. 1980/ Upper river | 1979 (1month) | Gambia | Sylvatic | Cases: 244  All ages | Retrospective case finding and surveillance | Death: 65  Attack rate: 0,04565  Incidence per 100000: 45,7  Case fatality rate (%): 26.6  Mortality per 100000: 12.2 | Hospital & field | Rural |
| Agadzi et al. 1984 Upper Volt | N/A | Ghana | Urban | Cases: 136  All ages | Surveillance | Death: 34  Case fatality rate (%):25 | Hospital | Rural |
| Agadzi et al. 1984 Eastern Region | N/A | Ghana | Urban | Cases: 207  All ages | Surveillance | Death: 44  Attack rate: 0,04565  Incidence per 100000: 10,350  Case fatality rate (%): 21,6  Mortality per 100000: 2200 | Hospital | Rural |
| Agadzi et al. 1984 Volta Region | N/A | Ghana | Urban | Cases: 340  All ages | Surveillance | Death: 52  Case fatality rate (%): 15.29 | Hospital | Rural |
| Agadzi et al. 1984 Brong Ahafo | N/A | Ghana | Urban | Cases: 104  All ages | Surveillance | Death: 44  Case fatality rate (%): 42.3% | Hospital | Rural |
| Otshudiema et al. 2017 | 2016(1month) | DR Congo | Sylvatic | Cases: 410  0 – 72 years | Case based surveillance | Death: 42  Attack rate: 0,00013  Incidence per 100000: 13  Case fatality rate (%): 10.2  Mortality per 100000: 1.3 | Hospital & field | Urban |
| Nasidi et al. 1989  Oyo | 1987 (N/A) | Nigeria | Urban | Cases: 805  All ages | Survey & case finding | Death: 416  Attack rate: 0,000111  Incidence per 100000: 11  Case fatality rate (%): 52  Mortality per 100000: 6 | Hospital & field | Urban |
| Nasidi et al. 1989  Ogun | 1987(N/A) | Nigeria | Urban | Cases: 72  All ages | Survey & case finding | Death: 8  Incidence per 100000: 2.6  Case fatality rate (%): 11  Mortality per 100000: 0.3 | Hospital & field | Urban |
| Nasidi et al. 1989  Ondo | 1987(N/A) | Nigeria | Urban | Cases: 9  All ages | Survey & case finding | Death: 3  Incidence per 100000: 0.3  Case fatality rate (%): 33  Mortality per 100000: 0.1 | Hospital & field | Urban |
| Nasidi et al. 1989  Kwara | 1987(N/A) | Nigeria | Urban | Cases: 7  All ages | Survey & case finding | Death: 6  Incidence per 100000: 0.2  Case fatality rate (%): 85  Mortality per 100000: 0.2 | Hospital & field | Urban |
| Nasidi et al. 1989  Lagos | 1987(N/A) | Nigeria | Urban | Cases: 69  All ages | Survey & case finding | Death: 9  Incidence per 100000: 1.4  Case fatality rate (%): 13  Mortality per 100000: 0.2 | Hospital & field | Urban |
| Social Services Imo State 1994 | 1994(3months) | Nigeria | N/A | Cases: 83  All ages | Survey & case finding | Death: 44  Attack rate: 0,000838  Incidence per 100000: 83,8  Case fatality rate (%): 53  Mortality per 100000: 44.4 | Hospital & field | Rural |
| De Cock et al. 1988/ Cross river | 1986(1month) | Nigeria | Sylvatic | Cases: 126  All ages | Epidemiological survey | Death: 59  Attack rate: 0,000838  Incidence per 100000: 63  Case fatality rate (%): 46,8  Mortality per 100000: | Hospital & field | Rural |

**Table S3: Risk of bias of assessment of included studies (adapted from Hoy et al, 2012)**

| **Study ID** | **Representation** | **Sampling** | **Random selection** | **Non response bias** | **Data collection** | **Case definition** | **Reliability of tool** | **Method of data collection** | **Prevalence period** | **Numerator and Denominators** | **Summary assessment** |
| --- | --- | --- | --- | --- | --- | --- | --- | --- | --- | --- | --- |
| Thonnon 1998a | 0 | 0 | 0 | 0 | 0 | 0 | 0 | 0 | 1 | 0 | 1 |
| Thonnon 1998b | 0 | 0 | 0 | 0 | 0 | 0 | 0 | 0 | 1 | 0 | 1 |
| Lilay et al. 2017 | 0 | 0 | 1 | 1 | 0 | 0 | 0 | 0 | 1 | 1 | 4 |
| Sanders et al. 1998 | 0 | 1 | 1 | 1 | 0 | 0 | 0 | 0 | 1 | 0 | 4 |
| Wamala et al. 2012 | 0 | 1 | 1 | 0 | 0 | 0 | 0 | 0 | 1 | 0 | 3 |
| Kwagonza et al. 2018 | 0 | 0 | 0 | 1 | 0 | 0 | 0 | 0 | 1 | 0 | 2 |
| Monath et al. 1980 | 0 | 1 | 1 | 1 | 0 | 0 | 0 | 0 | 1 | 0 | 4 |
| Agadzi et al. 1984 | 0 | 0 | 1 | 1 | 1 | 0 | 0 | 1 | 1 | 1 | 6 |
| Otshudiema et al. 2017 | 1 | 1 | 1 | 0 | 0 | 0 | 0 | 1 | 1 | 1 | 6 |
| Nasidi et al. 1989 | 0 | 1 | 1 | 1 | 0 | 0 | 0 | 0 | 1 | 0 | 4 |
| Social service Imo state 1994 | 0 | 0 | 0 | 0 | 0 | 0 | 0 | 0 | 1 | 0 | 1 |
| De Cock et al. 1988 | 0 | 0 | 1 | 1 | 1 | 0 | 0 | 1 | 1 | 0 | 5 |

Point scored: Low risk: 0-3; Moderate risk: 4-6 and High risk: 7-9
